# Supplementary material for: Uncertainty-aware single-cell annotation with a hierarchical reject option
Source: Bioinformatics. 2024 Mar 5;40(3):btae128. doi: 10.1093/bioinformatics/btae128 (PMC10957513; doi:10.1093/bioinformatics/btae128)
Supplement: btae128_Supplementary_Data [file btae128_supplementary_data.pdf]

1 implementation details

1.1 Hyperparameters

Inside the flat and hierarchical annotation models, the LinearSVC(), the LogisticRegression() and the RandomForests() classifiers of scikit-learn [Pedregosa et al., 2011] were implemented. The hyperparameters implemented for these classifiers were the following:

- for the RandomForests() classifier:
  - n_estimators: 500
  - random_state: 0
  - max_depth: 30, 50 ,None
- for the LinearSVC classifier:
  - penalty: l2
  - C: 1, 10, 1000, 10000, 100000, 1000000
- for the LogisticRegression() classifier:
  - multi_class: multinomial
  - penalty: l2
  - max_iter: 2000

Highly variable gene (HVG) selection is performed with scanpy under the 'seurat' setting [Satija et al., 2015, Wolf et al., 2018]. For the F-test feature selection, the features were ranked according to their F-statistics and the number of selected features is considered as a hyperparameter. The tested hyperparameters range for the HVG selection was: [500, 1000, 2000, 5000, 8000, 1000] and for F selection: [1000, 5000, 10000, 15000, 18000, 20000], though sometimes one or several parameters were excluded due to running time problems. A detailed spreadsheet can be obtained through contact with the authors.

1.2 Algorithms

The algorithms of greedy and non-greedy top-down label assignment and bottom-up label assignment are respectively defined in Algorithm S1, S2 and S3. Greedy and non-greedy label assignment (without rejection) corresponds to Algorithms S1, S2 when $t_r = 0$.

Algorithm 1 Top-down greedy label assignment

Input test instance $\mathbf{x}$, a trained hierarchical model, rejection threshold t_r

$v \leftarrow Root$, $P_{path} = 1$
while v is not a leaf **do**
    $P_{opt}(\mathbf{x}) = 0$, $v_{opt} \leftarrow v$
    for v' in children(v) **do**
        if $P(y \in v'|v, \mathbf{x}) > P_{opt}(\mathbf{x})$ **then**
            $P_{opt}(\mathbf{x}) \leftarrow P(y \in v'|v, \mathbf{x})$
            $v_{opt} \leftarrow v'$
        end if
    end for
    $v \leftarrow v_{opt}$
    $P_{path}(\mathbf{x}) \leftarrow P_{path}(\mathbf{x}) \times P_{opt}(\mathbf{x})$
    if $P_{path}(\mathbf{x}) < t_r$ **then**
        break
    end if
end while
return $v, P_{path}(\mathbf{x})$

Algorithm 2 Top-down non-greedy label assignment

Input test instance $\mathbf{x}$, a trained hierarchical model, rejection threshold t_r

$Q \leftarrow$ Priority queue
$Q.addCouple(1.0, Root)$
while $Q \neq \emptyset$ **do**
    $(P(\mathbf{x}), v) \leftarrow$ Q.popTopCouple()
    if $P(\mathbf{x}) < t_r$ or v is a leaf **then**
        break
    end if
    for v' in $children$(v) **do**
        $P'(\mathbf{x}) \leftarrow P(y \in v'|v, \mathbf{x})$
        $Q.addCouple(P(\mathbf{x}) \times P'(\mathbf{x}), v')$
    end for
end while
return $v, P(\mathbf{x})$

Algorithm 3 Bottom-up label assignment

Input test instance $\mathbf{x}$, flat probabilistic classifier with associated prediction probability vector $P(y = c \,|\, \mathbf{x})$ for corresponding classes $\{c_1, ..., c_K\}$, rejection threshold t_r, a hierarchy T

$\mathcal{L} \leftarrow$ set that contains all leaves $\widehat{=}\{c_1, ..., c_K\}$
$v_{max} \leftarrow$ leaf that maximizes $P(y = c \,|\, \mathbf{x})$
$p_{max} \leftarrow \max_c(P(y = c \,|\, \mathbf{x}))$
while $p_{max} < t_r \wedge v_{max} \neq$ root **do**
    $\mathcal{L} \leftarrow$ all parents of nodes in $\mathcal{L}$
    for $v \in \mathcal{L}$ **do**
        compute $P(y \in v|\mathbf{x})$ using Eqn. (3)
    end for
    $v_{max} \leftarrow$ parent of v_{max}
    $p_{max} \leftarrow P(y \in v_{max}|\mathbf{x})$
end while
return v_{max}, p_{max}

2 Supplementary tables

Dataset	N° cells	N° genes	N° cell types	Protocol	Description	Balanced hierarchy	Label levels	Reference
AMB	12 822	45 625	3/16/92*	SMART-seq v4	Primary mouse visual cortex	Yes	3	Tasic et al. [2018]
COVID	15 725	24 444	41	NovaSeq v4	BALF	No	2-5	Chan Zuckerberg Initiative Single-Cell COVID-19 Consortia et al. [2020]
Azimuth PBMC	160 698	20 729	55**	CITE-seq	Human bone marrow	No	2-5	Stuart et al. [2019]
Flyhead	53 809	13 056	71**	10X	Head of the fruit fly	No	3-9	Li et al. [2022]
Flybody	88 754	15 267	30**	10X	Body of the fruit fly	No	3-8	Li et al. [2022]
PbmcBench: pbmc1_10Xv2	6444	33694	9**	10X version 2	PBMC	No	1-3	Ding et al. [2020]
PbmcBench: pbmc1_CL	253	33694	7**	CEL-Seq2	PBMC	No	1-3	Ding et al. [2020]
PbmcBench: pbmc1_DR	3222	33694	9**	Drop-Seq	PBMC	No	1-3	Ding et al. [2020]
PbmcBench: pbmc1_iD	3222	33694	7**	inDrop	PBMC	No	1-3	Ding et al. [2020]
PbmcBench: pbmc1_SM2	253	33694	6**	SMART-Seq2	PBMC	No	1-3	Ding et al. [2020]
PbmcBench: pbmc1_SW	3176	33694	7**	Seq-Well	PBMC	No	1-3	Ding et al. [2020]
PbmcBench: pbmc2_10Xv2	3362	33694	9**	10Xv2	PBMC	No	1-3	Ding et al. [2020]

Table 1: Characteristics of the datasets used in this study after preprocessing (see Section 2.1).
* Number of cell type populations in every level of the hierarchy.
** Number of cell type populations at the lowest level of the hierarchy, for unbalanced datasets this lowest level is defined as the last sublabel available for every specific cell type population.

		AMB	COVID	Azimuth PBMC	Flyhead	Flybody
Greedy	LR	0.927	0.806	0.910	0.917	0.956
	RF	0.853	0.686	0.798	0.832	0.928
	SVM	0.917	0.783	0.891	0.914	0.953
Non-Greedy	LR	0.927	0.806	0.904	0.919	0.955
	RF	0.841	0.689	0.799	0.832	0.928
	SVM	0.856	0.731	0.515	0.462	0.797

Table 2: The average accuracy scores across the five cross-validation folds of greedy and non greedy hierarchical annotation across the AMB, COVID, Azimuth PBMC, Flyhead and Flybody dataset for the logistic regression(LR), random forests(RF) and linear SVM classifier.

		AMB	COVID	Azimuth PBMC	Flyhead	Flybody
Greedy	LR	0.835	0.594	0.803	0.831	0.829
	RF	0.660	0.329	0.481	0.628	0.562
	SVM	0.805	0.556	0.788	0.819	0.821
Non-Greedy	LR	0.834	0.594	0.788	0.829	0.823
	RF	0.662	0.343	0.488	0.628	0.562
	SVM	0.714	0.471	0.595	0.211	0.588

Table 3: The balanced accuracy scores of greedy and non greedy hierarchical annotation across the AMB, COVID, Azimuth PBMC, Flyhead and Flybody dataset for the logistic regression (LR), random forests (RF) and linear SVM classifier.

3 Supplementary figures

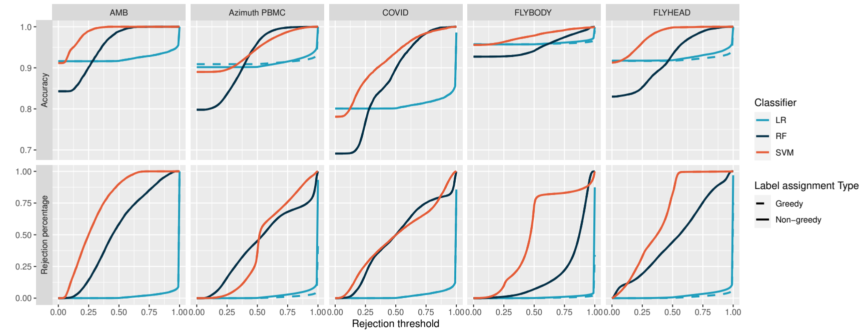

Figure 1: Accuracy-rejection curves of hierarchical annotation with greedy and non-greedy prediction of the AMB, Azimuth PBMC, COVID, Flybody and Flyhead datasets with Logistic regression (LR), Random forests (RF) and linear SVM (SVM) classifiers. The curves in the top row represent the accuracy score of the non-rejected label given a rejection threshold value. The bottom row curves represent the percentage of rejected labels for the rejection threshold value.

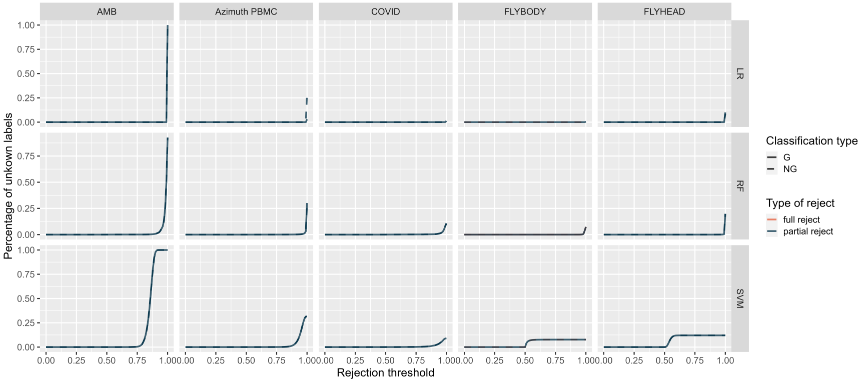

Figure 2: A comparison of the percentage of unknown labels assigned during the annotation task when partial and full rejection is implemented during greedy and non-greedy hierarchical annotation. Evaluation is performed with three classifiers (logistic regression, random forests and linear SVM) across 5 datasets (AMB, Azimuth PBMC, COVID, Flyhead and Flybody).

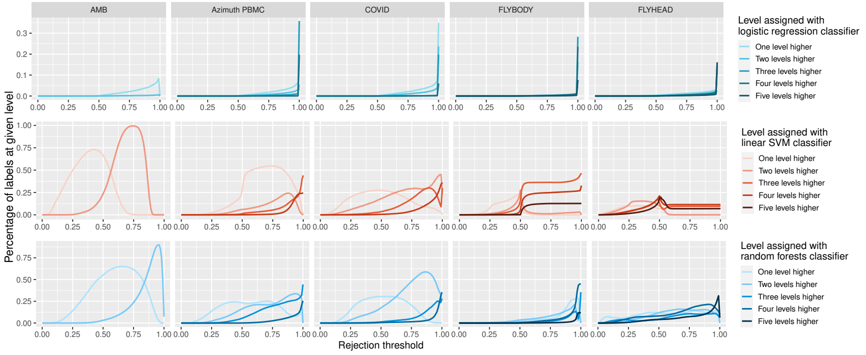

Figure 3: Details of partial rejection performed with non-greedy hierarchical annotation across a rejection threshold range from 0.0 to 1.0 for three classifiers (logistic regression - light blue, linear SVM - red, random forests - dark blue) across 5 datasets (AMB, Azimuth PBMC, COVID, Flyhead and Flybody). For all of these analyses, the percentage of labels that are assigned one level higher than the full label, two levels higher etc. is visualized.

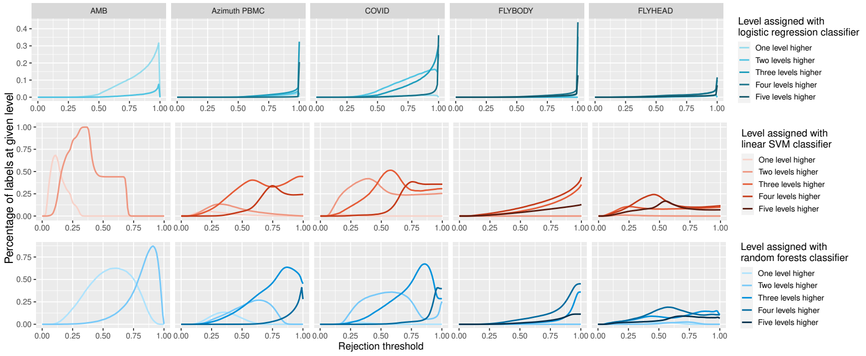

Figure 4: Details of partial rejection performed with flat annotation across a rejection threshold range from 0.0 to 1.0 for three classifiers (logistic regression - light blue, linear SVM - red, random forests - dark blue) across 5 datasets (AMB, Azimuth PBMC, COVID, Flyhead and Flybody). For all of these analyses, the percentage of labels that are assigned one level higher than the full label, two levels higher etc. is visualized.

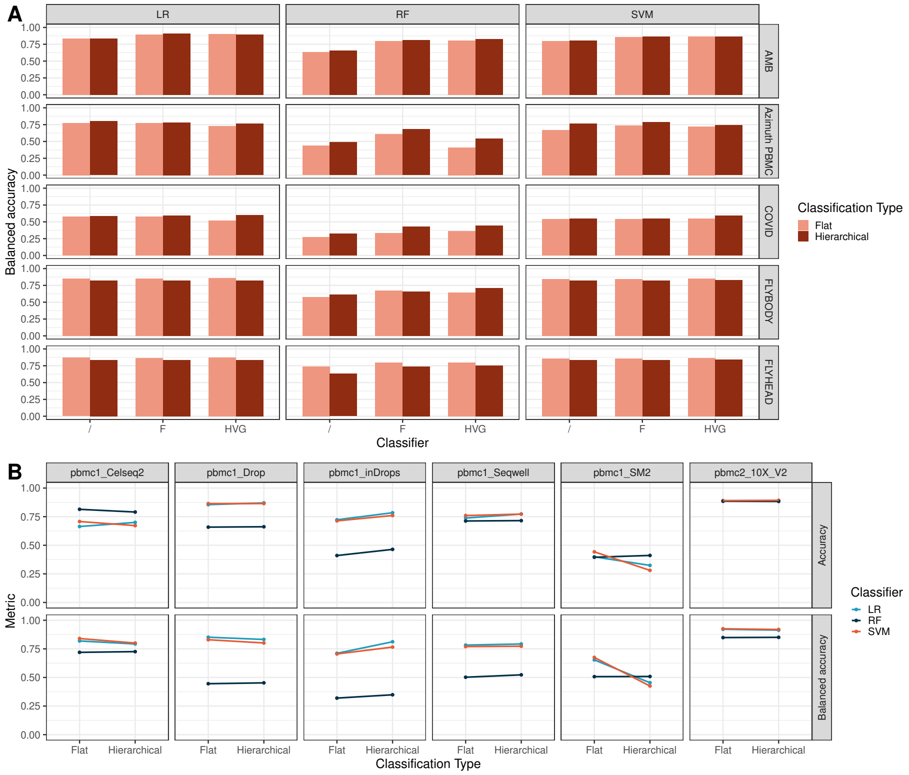

Figure 5: Flat versus hierarchical annotation performance without rejection on intra- and interdataset analyses. **A.** Comparison of the overall intradataset performance of flat versus hierarchical classification in terms of the balanced accuracy score for the AMB, AzimuthPBMC and COVID dataset (Table S1) with the use of logistic regression classifiers (LR), random forest classifiers (RF) and linear support vector machine classifiers (SVM). The classifiers are implemented with three feature selection strategies: no feature selection (/), the F-test (F) and highly variable gene selection (HVG). **B.** Interdataset performance of flat and hierarchical classification across the three classifiers without feature selection in terms of the accuracy and balanced accuracy score. The classifiers are trained on the pbmc1 sample, sequenced with the 10Xv2 technology. Performance is tested on sequenced data of the same sample across different plaforms and on a second biological sample (pbmc2), sequenced on the same platform.

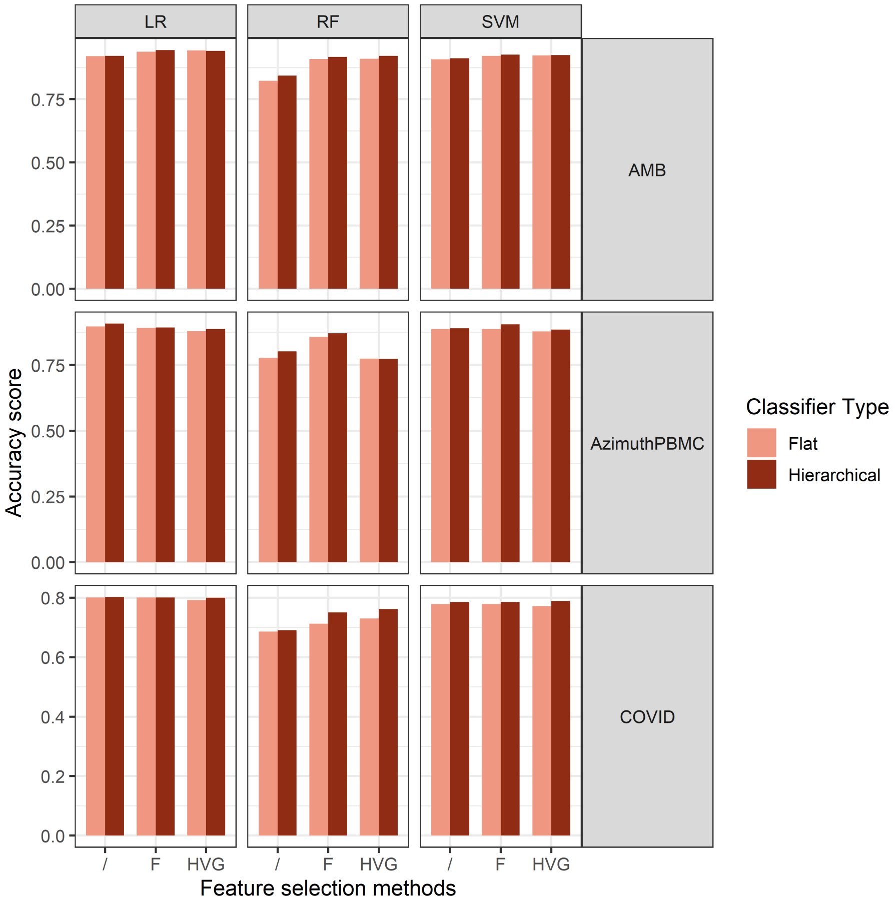

Figure 6: Comparison of the overall intradataset performance of flat versus hierarchical classification in terms of the accuracy score for the AMB, Azimuth-PBMC and COVID dataset (Table S1) with the use of logistic regression classifiers (LR), random forest classifiers (RF) and linear support vector machine classifiers (SVM). The classifiers are implemented with three feature selection strategies: no feature selection (/), the F-test (F) and highly variable gene selection (HVG).

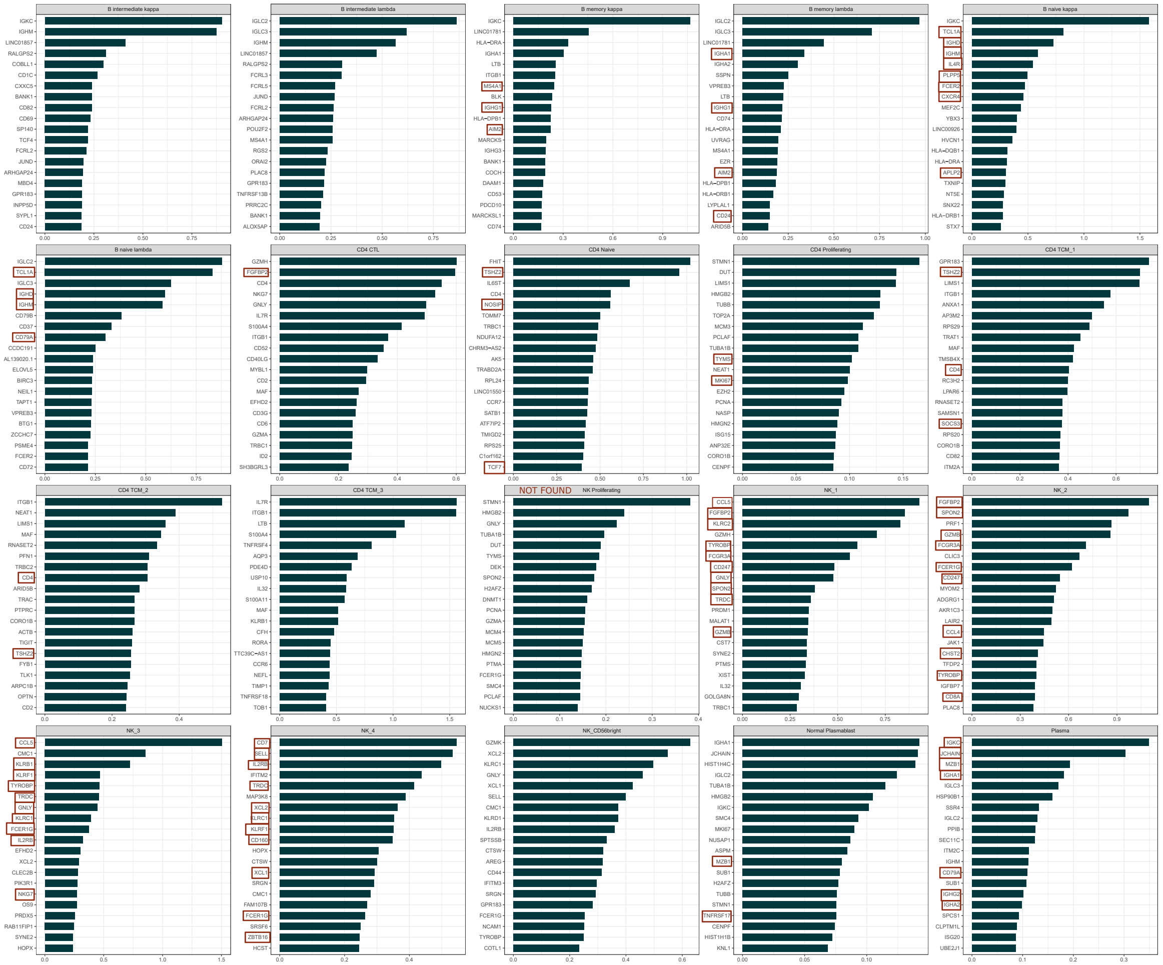

Figure 7: Part 1. Visualization of the top-twenty (positive) coefficients of flat annotation with logistic regression on the AzimuthPBMC dataset that leads to the final label classes. If the genes of these coefficients correspond to marker genes their names on the y-axis are enclosed by a red box. The coefficients of the major cell type classes are visualised in light blue. If marker genes were not found for the cell (sub)types this is indicated by the words "NOT FOUND" in red in the grey square depicting the subtype's name.

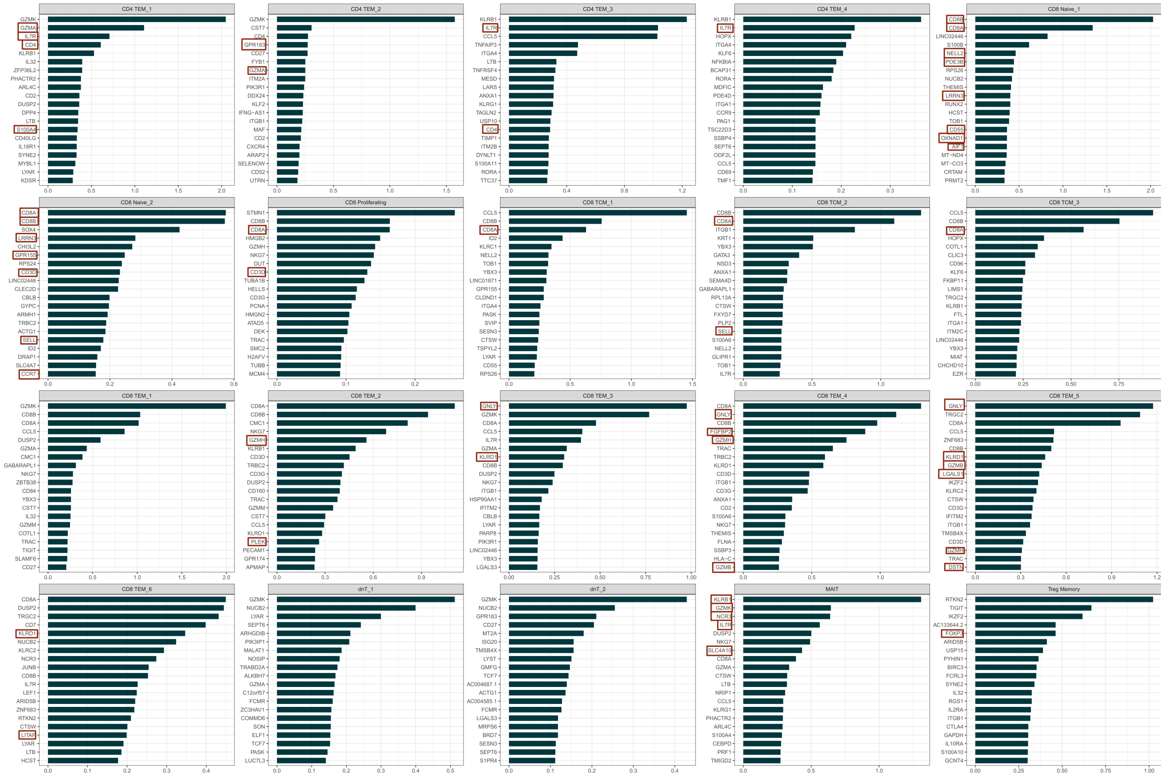

Figure 8: Part 2. Visualization of the top-twenty (positive) coefficients of flat annotation with logistic regression on the AzimuthPBMC dataset that leads to the final label classes. If the genes of these coefficients correspond to marker genes their names on the y-axis are enclosed by a red box. The coefficients of the major cell type classes are visualised in light blue. If marker genes were not found for the cell (sub)types this is indicated by the words "NOT FOUND" in red in the grey square depicting the subtype's name.

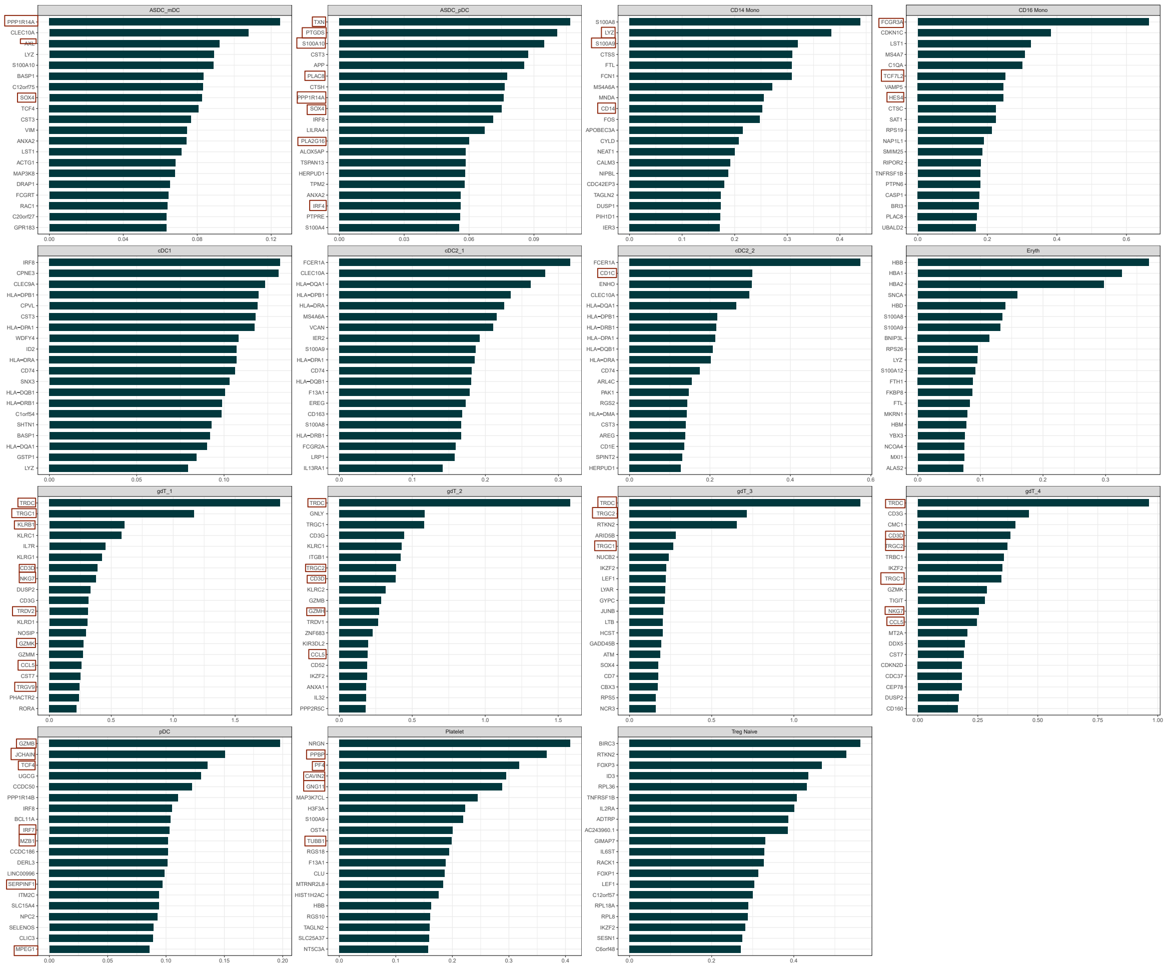

Figure 9: Part 3. Visualization of the top-twenty (positive) coefficients of flat annotation with logistic regression on the AzimuthPBMC dataset that lead to the final label classes. If the genes of these coefficients correspond to marker genes their names on the y-axis are enclosed by a red box. The coefficients of the major cell type classes are visualised in light blue. If marker genes were not found for the cell (sub)types this is indicated by the words "NOT FOUND" in red in the grey square depicting the subtype's name.

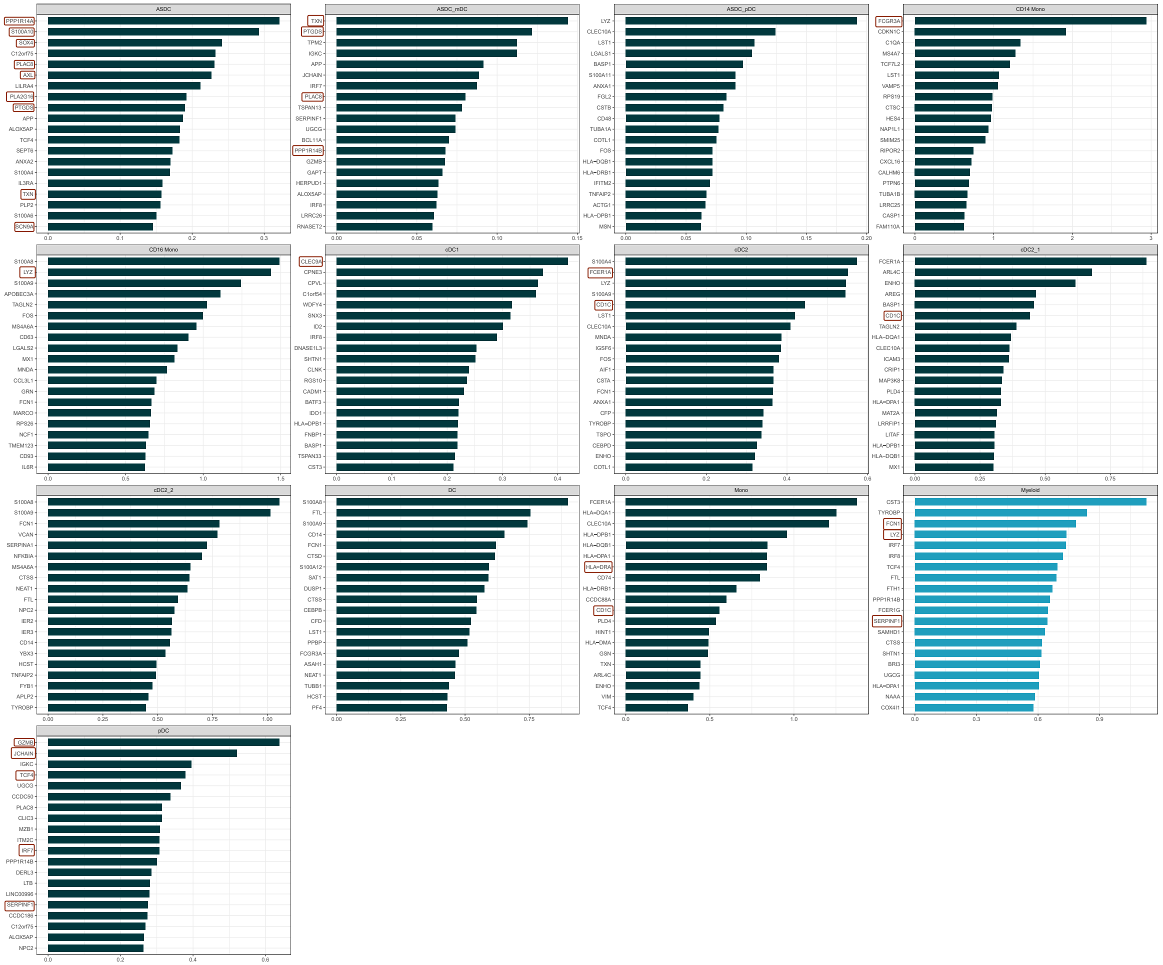

Figure 10: Visualization of the top-twenty (positive) coefficients of all myeloid sub- and super-labels for non-greedy hierarchical annotation with logistic regression on the AzimuthPBMC dataset that leads to the final label classes. If the genes of these coefficients correspond to marker genes their names on the y-axis are enclosed by a red box. The coefficients of the major cell type classes are visualised in light blue. If marker genes were not found for the cell (sub)types this is indicated by the words "NOT FOUND" in red in the grey square depicting the subtype's name.

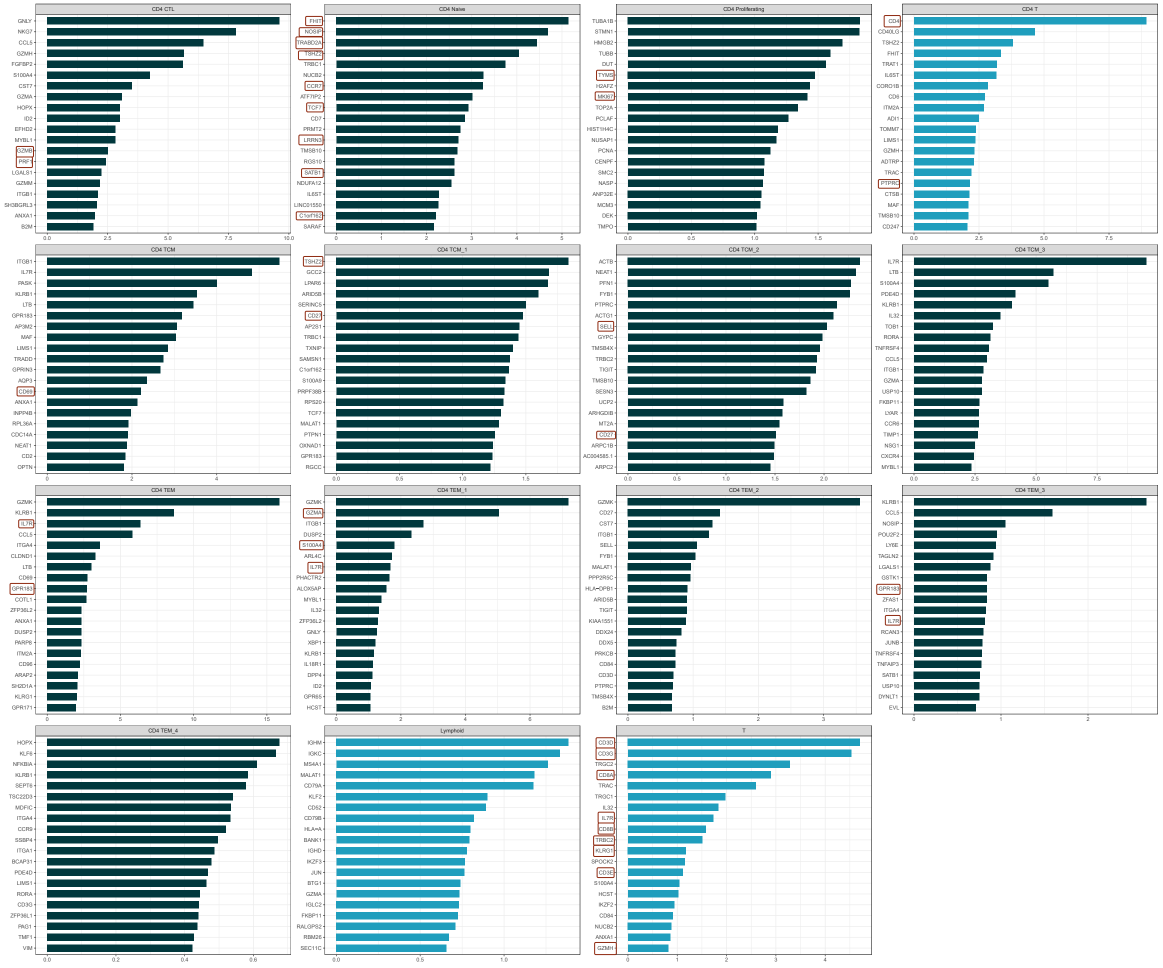

Figure 11: Visualization of the top-twenty (positive) coefficients of all CD4+ T cell sub- and super-labels for non-greedy hierarchical annotation with logistic regression on the AzimuthPBMC dataset that leads to the final label classes. If the genes of these coefficients correspond to marker genes their names on the y-axis are enclosed by a red box. The coefficients of the major cell type classes are visualised in light blue. If marker genes were not found for the cell (sub)types this is indicated by the words "NOT FOUND" in red in the grey square depicting the subtype's name.

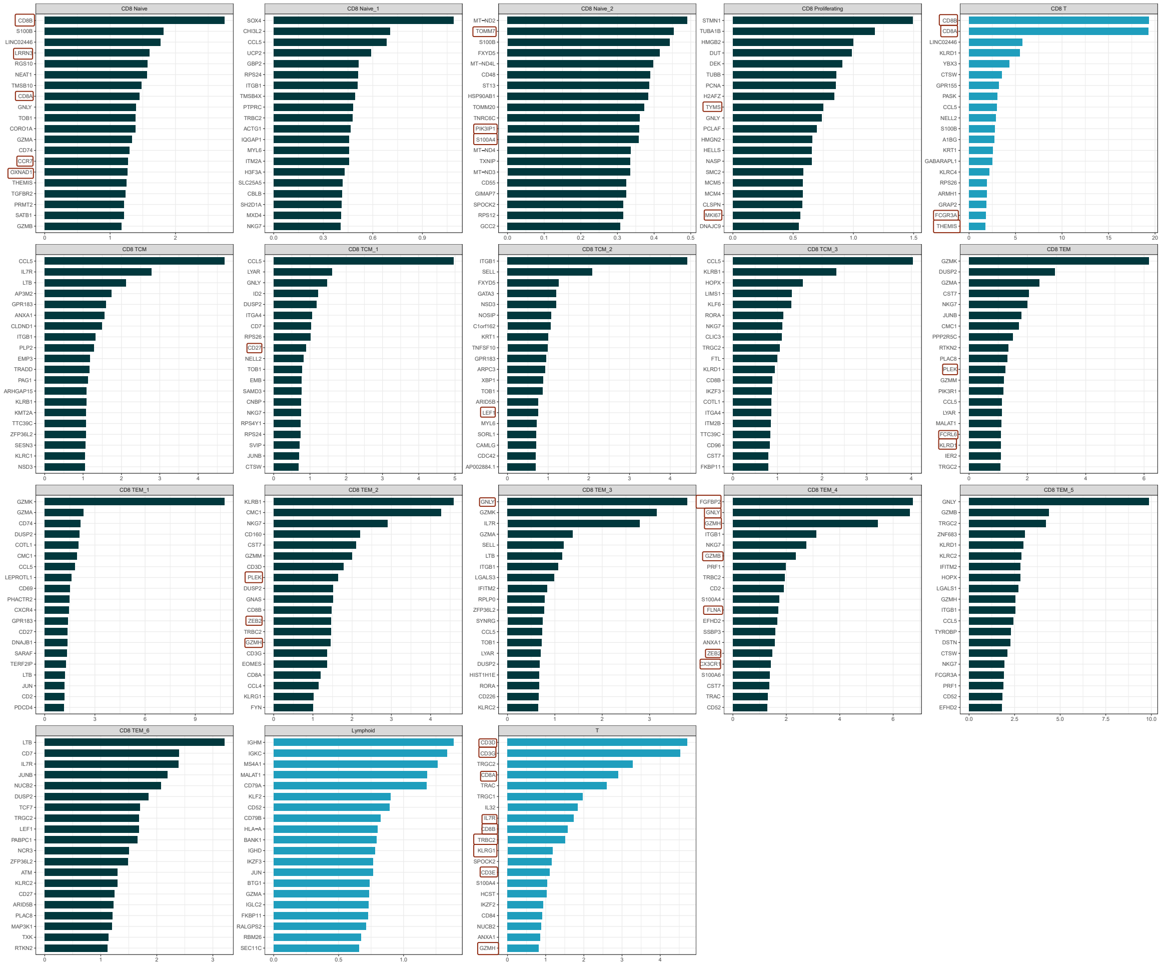

Figure 12: Visualization of the top-twenty (positive) coefficients of all CD8+ T cell sub- and super-labels for non-greedy hierarchical annotation with logistic regression on the AzimuthPBMC dataset that leads to the final label classes. If the genes of these coefficients correspond to marker genes their names on the y-axis are enclosed by a red box. The coefficients of the major cell type classes are visualised in light blue. If marker genes were not found for the cell (sub)types this is indicated by the words "NOT FOUND" in red in the grey square depicting the subtype's name.

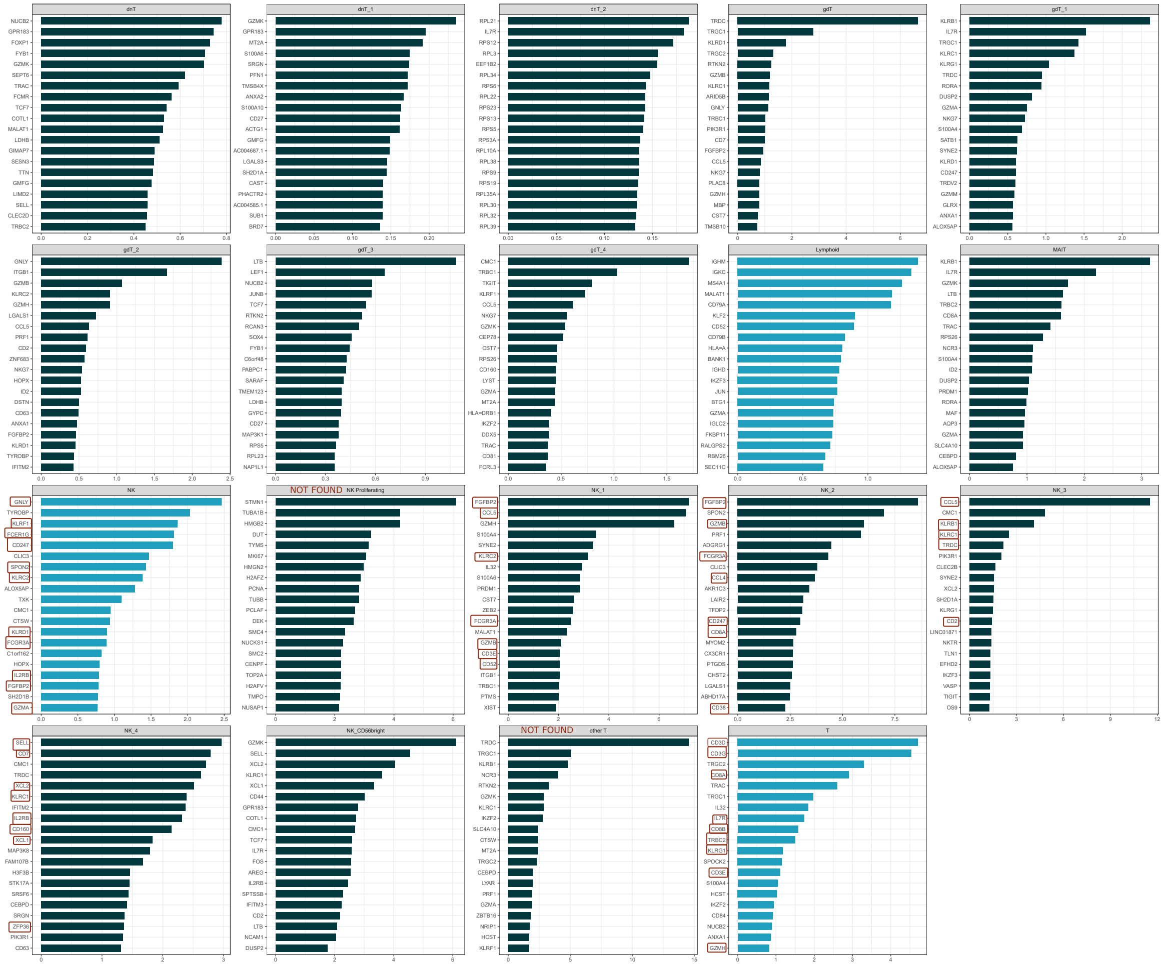

Figure 13: Visualization of the top-twenty (positive) coefficients of all other T and NK cell sub- and super-labels for non-greedy hierarchical annotation with logistic regression on the AzimuthPBMC dataset that leads to the final label classes. If the genes of these coefficients correspond to marker genes their names on the y-axis are enclosed by a red box. The coefficients of the major cell type classes are visualised in light blue. If marker genes were not found for the cell (sub)types this is indicated by the words "NOT FOUND" in red in the grey square depicting the subtype's name.

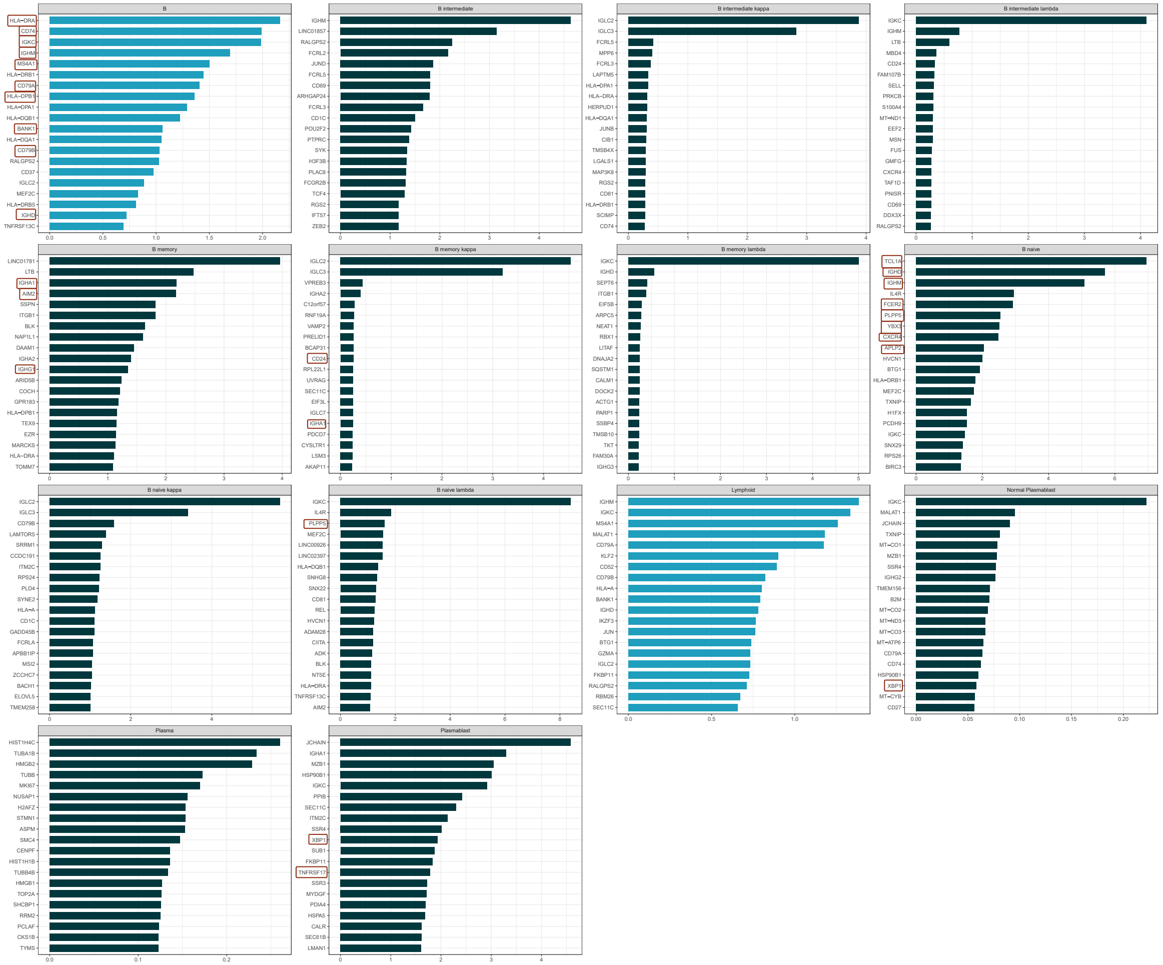

Figure 14: Visualization of the top-twenty (positive) coefficients of all other B cell sub- and super-labels for non-greedy hierarchical annotation with logistic regression on the AzimuthPBMC dataset that leads to the final label classes. If the genes of these coefficients correspond to marker genes their names on the y-axis are enclosed by a red box. The coefficients of the major cell type classes are visualised in light blue. If marker genes were not found for the cell (sub)types this is indicated by the words "NOT FOUND" in red in the grey square depicting the subtype's name.

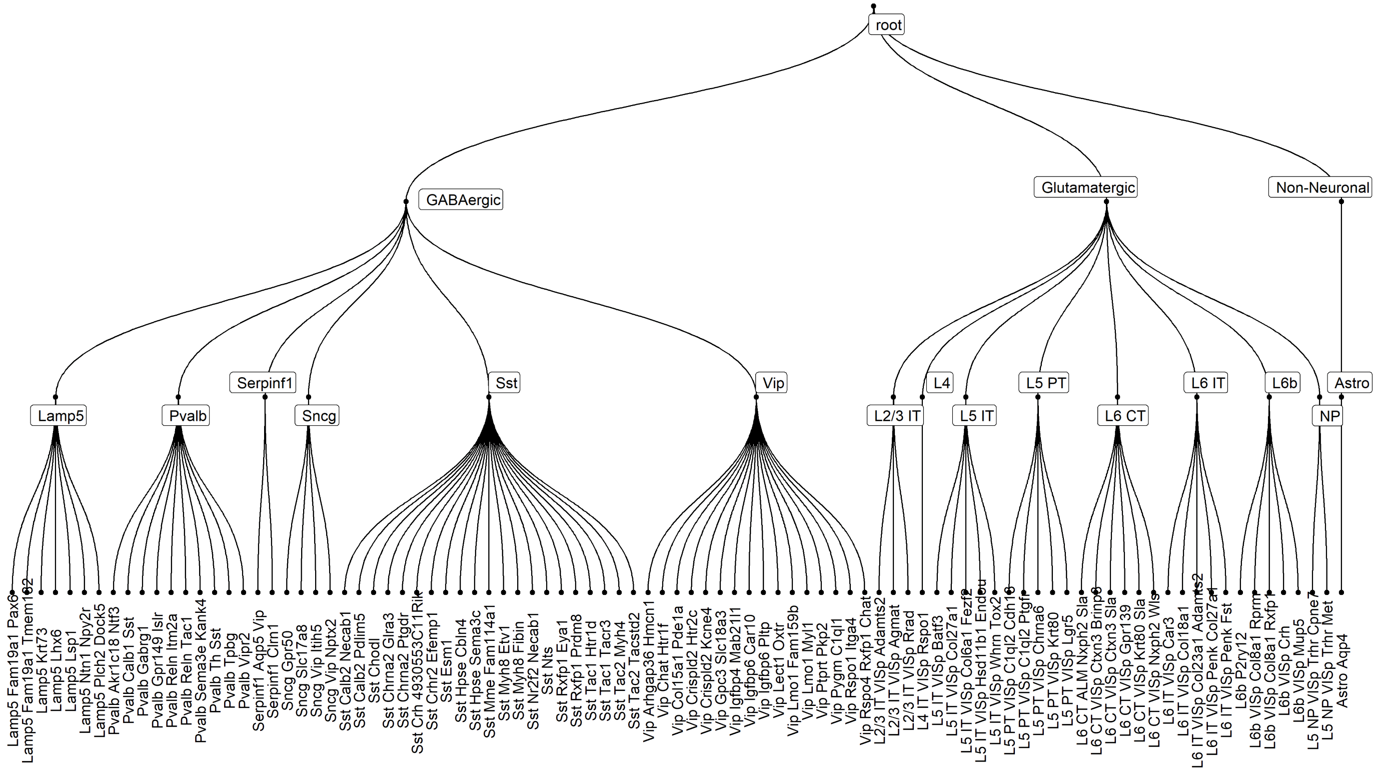

Figure 15: Visualisation of the hierarchy of the AMB dataset.

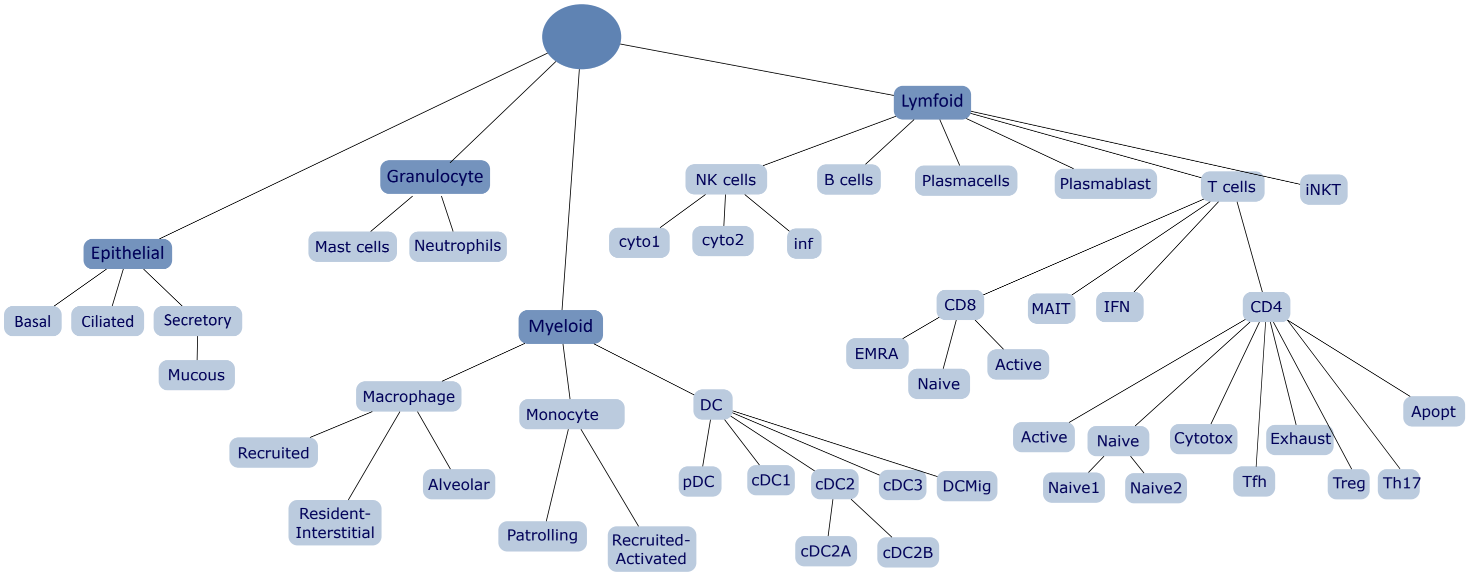

Figure 16: Visualisation of the hierarchy of the COVID dataset.

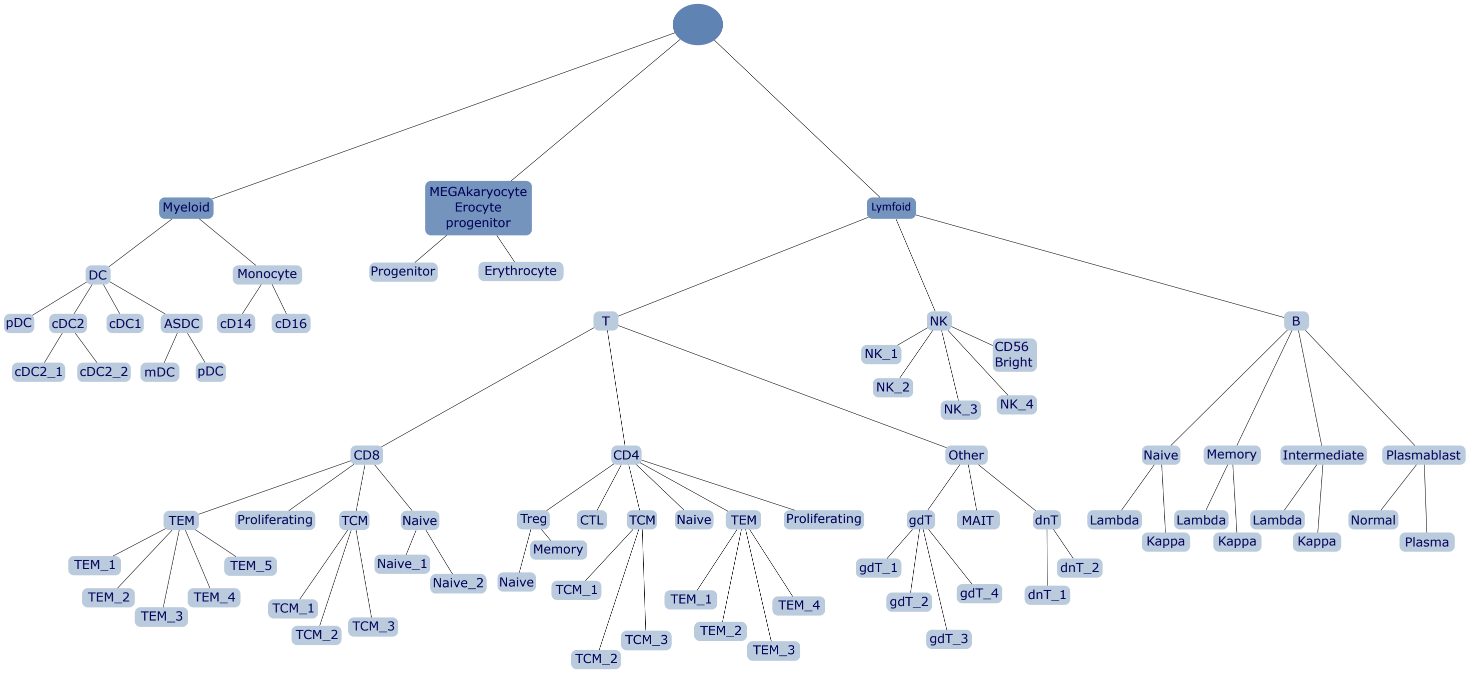

Figure 17: Visualisation of the hierarchy of the Azimuth PBMC dataset.

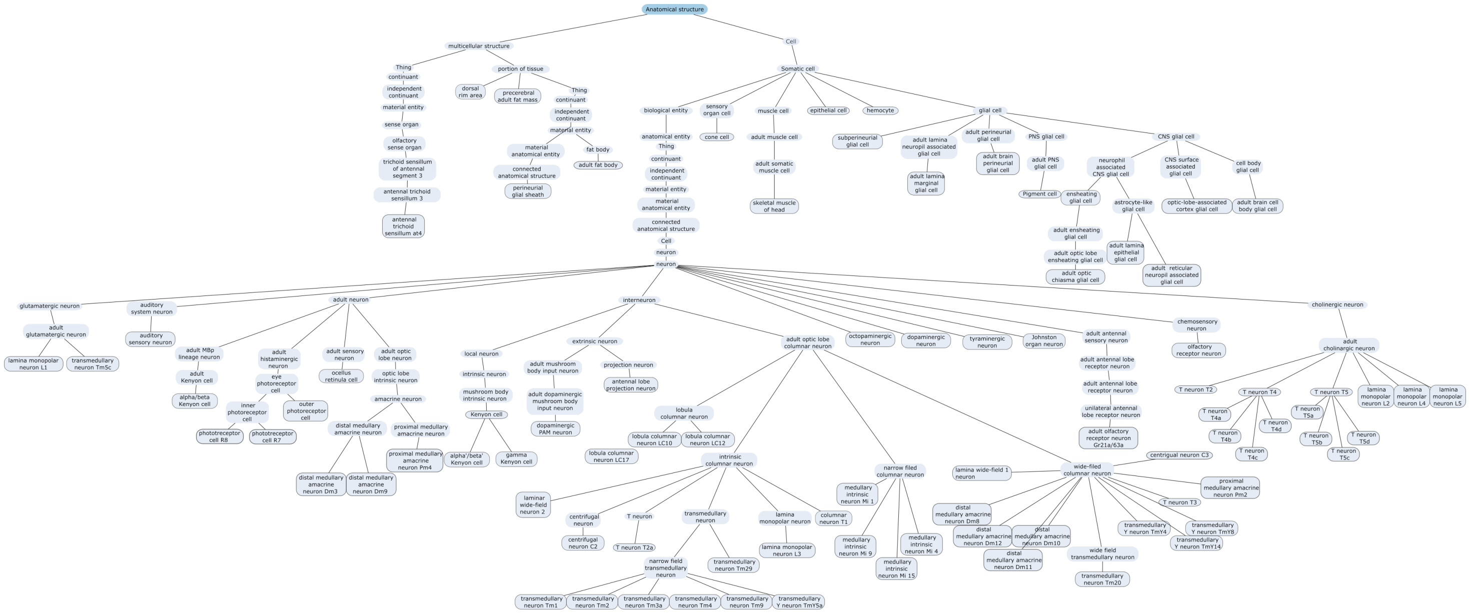

Figure 18: Visualisation of the hierarchy of the Flyhead dataset prior to exclusion of the uninformative labels.

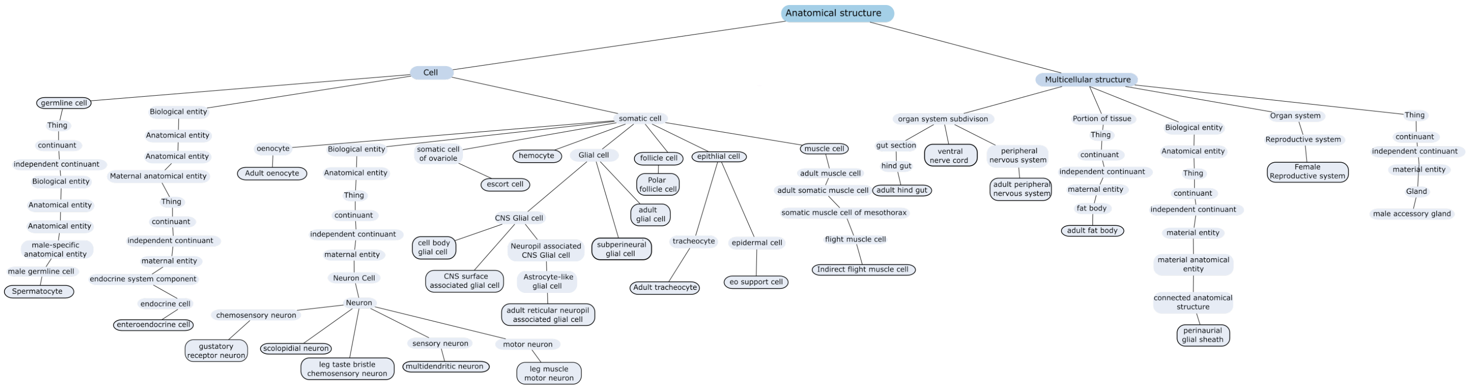

Figure 19: Visualisation of the hierarchy of the Flybody dataset prior to exclusion of the uninformative labels.

References

Chan Zuckerberg Initiative Single-Cell COVID-19 Consortia, E. Ballestar, D. L. Farber, S. Glover, B. Horwitz, K. Meyer, M. Nikolić, J. Ordovas-Montanes, P. Sims, A. Shalek, N. Vandamme, L. Vandekerckhove, R. Vento-Tormo, and A. C. Villani. Single cell profiling of COVID-19 patients: an international data resource from multiple tissues. preprint, Infectious Diseases (except HIV/AIDS), Nov. 2020. URL http://medrxiv.org/lookup/doi/10.1101/2020.11.20.20227355.

J. Ding, X. Adiconis, S. K. Simmons, M. S. Kowalczyk, C. C. Hession, N. D. Marjanovic, T. K. Hughes, M. H. Wadsworth, T. Burks, L. T. Nguyen, J. Y. H. Kwon, B. Barak, W. Ge, A. J. Kedaigle, S. Carroll, S. Li, N. Hacohen, O. Rozenblatt-Rosen, A. K. Shalek, A.-C. Villani, A. Regev, and J. Z. Levin. Systematic comparison of single-cell and single-nucleus RNA-sequencing methods. *Nature Biotechnology*, 38(6): 737–746, 2020. ISSN 1546-1696. doi: 10.1038/s41587-020-0465-8. URL https://www.nature.com/articles/s41587-020-0465-8.

H. Li, J. Janssens, M. De Waegeneer, S. S. Kolluru, K. Davie, V. Gardeux, W. Saelens, F. P. A. David, M. Brbić, K. Spanier, J. Leskovec, C. N. McLaughlin, Q. Xie, R. C. Jones, K. Brueckner, J. Shim, S. G. Tattikota, F. Schnorrer, K. Rust, T. G. Nystul, Z. Carvalho-Santos, C. Ribeiro, S. Pal, S. Mahadevaraju, T. M. Przytycka, A. M. Allen, S. F. Goodwin, C. W. Berry, M. T. Fuller, H. White-Cooper, E. L. Matunis, S. DiNardo, A. Galenza, L. E. O'Brien, J. A. T. Dow, FCA Consortium§, H. Jasper, B. Oliver, N. Perrimon, B. Deplancke, S. R. Quake, L. Luo, S. Aerts, D. Agarwal, Y. Ahmed-Braimah, M. Arbeitman, M. M. Ariss, J. Augsburger, K. Ayush, C. C. Baker, T. Banisch, K. Birker, R. Bodmer, B. Bolival, S. E. Brantley, J. A. Brill, N. C. Brown, N. A. Buehner, X. T. Cai, R. Cardoso-Figueiredo, F. Casares, A. Chang, T. R. Clandinin, S. Crasta, C. Desplan, A. M. Detweiler, D. B. Dhakan, E. Donà, S. Engert, S. Floc'hlay, N. George, A. J. González-Segarra, A. K. Groves, S. Gumbin, Y. Guo, D. E. Harris, Y. Heifetz, S. L. Holtz, F. Horns, B. Hudry, R.-J. Hung, Y. N. Jan, J. S. Jaszczak, G. S. X. E. Jefferis, J. Karkanias, T. L. Karr, N. S. Katheder, J. Kezos, A. A. Kim, S. K. Kim, L. Kockel, N. Konstantinides, T. B. Kornberg, H. M. Krause, A. T. Labott, M. Laturney, R. Lehmann, S. Leinwand, J. Li, J. S. S. Li, K. Li, K. Li, L. Li, T. Li, M. Litovchenko, H.-H. Liu, Y. Liu, T.-C. Lu, J. Manning, A. Mase, M. Matera-Vatnick, N. R. Matias, C. E. McDonough-Goldstein, A. McGeever, A. D. McLachlan, P. Moreno-Roman, N. Neff, M. Neville, S. Ngo, T. Nielsen, C. E. O'Brien, D. Osumi-Sutherland, M. N. Özel, I. Papatheodorou, M. Petkovic, C. Pilgrim, A. O. Pisco, C. Reisenman, E. N. Sanders, G. Dos Santos, K. Scott, A. Sherlekar, P. Shiu, D. Sims, R. V. Sit, M. Slaidina, H. E. Smith, G. Sterne, Y.-H. Su, D. Sutton, M. Tamayo, M. Tan, I. Tastekin, C. Treiber, D. Vacek, G. Vogler, S. Waddell, W. Wang, R. I. Wilson, M. F. Wolfner, Y.-C. E. Wong, A. Xie, J. Xu, S. Yamamoto, J. Yan, Z. Yao, K. Yoda, R. Zhu, and R. P. Zinzen. Fly Cell Atlas: A single-nucleus

transcriptomic atlas of the adult fruit fly. *Science (New York, N.Y.)*, 375 (6584):eabk2432, Mar. 2022. ISSN 1095-9203. doi: 10.1126/science.abk2432.

F. Pedregosa, G. Varoquaux, A. Gramfort, V. Michel, B. Thirion, O. Grisel, M. Blondel, P. Prettenhofer, R. Weiss, V. Dubourg, J. Vanderplas, A. Passos, D. Cournapeau, M. Brucher, M. Perrot, and E. Duchesnay. Scikit-learn: Machine learning in Python. *Journal of Machine Learning Research*, 12: 2825–2830, 2011.

R. Satija, J. A. Farrell, D. Gennert, A. F. Schier, and A. Regev. Spatial reconstruction of single-cell gene expression data. *Nature Biotechnology*, 33(5):495–502, 2015. ISSN 1546-1696. doi: 10.1038/nbt.3192. URL https://www.nature.com/articles/nbt.3192.

T. Stuart, A. Butler, P. Hoffman, C. Hafemeister, E. Papalexi, W. M. Mauck, Y. Hao, M. Stoeckius, P. Smibert, and R. Satija. Comprehensive Integration of Single-Cell Data. *Cell*, 177(7):1888–1902.e21, June 2019. ISSN 0092-8674. doi: 10.1016/j.cell.2019.05.031. URL https://www.sciencedirect.com/science/article/pii/S0092867419305598.

B. Tasic, Z. Yao, L. T. Graybuck, K. A. Smith, T. N. Nguyen, D. Bertagnolli, J. Goldy, E. Garren, M. N. Economo, S. Viswanathan, O. Penn, T. Bakken, V. Menon, J. Miller, O. Fong, K. E. Hirokawa, K. Lathia, C. Rimorin, M. Tieu, R. Larsen, T. Casper, E. Barkan, M. Kroll, S. Parry, N. V. Shapovalova, D. Hirschstein, J. Pendergraft, H. A. Sullivan, T. K. Kim, A. Szafer, N. Dee, P. Groblewski, I. Wickersham, A. Cetin, J. A. Harris, B. P. Levi, S. M. Sunkin, L. Madisen, T. L. Daigle, L. Looger, A. Bernard, J. Phillips, E. Lein, M. Hawrylycz, K. Svoboda, A. R. Jones, C. Koch, and H. Zeng. Shared and distinct transcriptomic cell types across neocortical areas. *Nature*, 563(7729): 72–78, Nov. 2018. ISSN 1476-4687. doi: 10.1038/s41586-018-0654-5. URL https://www.nature.com/articles/s41586-018-0654-5. Number: 7729 Publisher: Nature Publishing Group.

F. A. Wolf, P. Angerer, and F. J. Theis. SCANPY: Large-scale single-cell gene expression data analysis. *Genome Biology*, 19(1):15, 2018. ISSN 1474-760X. doi: 10.1186/s13059-017-1382-0. URL https://doi.org/10.1186/s13059-017-1382-0.
